# Pyrotinib Plus Vinorelbine *Versus* Lapatinib Plus Capecitabine in Patients With Previously Treated HER2-Positive Metastatic Breast Cancer: A Multicenter, Retrospective Study

**DOI:** 10.3389/fonc.2021.699333

**Published:** 2021-08-05

**Authors:** Yizhao Xie, Yi Li, Luo Ting, Die Sang, Peng Yuan, Wei Li, Huihui Li, Rui Ge, Biyun Wang

**Affiliations:** ^1^Department of Medical Oncology, Fudan University Shanghai Cancer Center, Shanghai, China; ^2^Department of Oncology, Shanghai Medical College, Fudan University, Shanghai, China; ^3^Guangzhou First People’s Hospital, School of Medicine, South China University of Technology, Guangzhou, China; ^4^Department of Head, Neck and Mammary Gland Oncology, Cancer Center, West China Hospital, Sichuan University, Sichuan, China; ^5^Department of Oncology, San Huan Cancer Hospital, Beijing, China; ^6^National Cancer Center, Tumor Hospital of the Chinese Academy of Medical Sciences, Beijing, China; ^7^Department of Medical Oncology, Jiangsu Province Hospital, Nanjing, China; ^8^Department of Breast Medical Oncology, Shandong Cancer Hospital and Institute, Shandong First Medical University and Shandong Academy of Medical Sciences, Jinan, China; ^9^Department of General Surgery, Huadong Hospital Affiliated to Fudan University, Shanghai, China

**Keywords:** breast cancer, HER2, pyrotinib, metastatic, combined therapy

## Abstract

**Background:**

Pyrotinib is a newly-developed irreversible pan-ErbB (erythroblastic leukemia viral oncogene homolog) receptor oral tyrosine kinase inhibitor (TKI) with promising efficacy in the human epidermal growth factor receptor-2 (HER2) positive breast cancer. The phase III PHOEBE study proved that pyrotinib plus capecitabine exceeded lapatinib plus capecitabine (LX) in PFS (p < 0.001). Oral vinorelbine is commonly used in combination with anti-HER2 treatment. However, no evidence was reported in terms of the real-world pattern, safety, and efficacy of pyrotinib plus vinorelbine (NP) compared with LX.

**Methods:**

Medical records were retrospectively evaluated for all HER2-positive metastatic breast cancer (MBC) patients who experienced progression on prior trastuzumab-containing regimens (advanced setting) and taxane (any setting) and received NP or LX therapy from 2015 to 2021 in five institutions.

**Results:**

A total of 224 patients were enrolled and evaluated, of which 132 (58.9%) patients received LX and 92 (41.1%) patients received NP. The median progression-free survival (mPFS) of NP group was significantly longer than that in LX group (8.3 *vs* 5.0 months, HR = 0.47 95% CI 0.34–0.65, p < 0.001). The advantage of NP over LX was seen both in patients with trastuzumab resistance (p < 0.001) and refractoriness (p = 0.004). The NP group had more diarrhea cases (23.9%) compared to the LX group (8.3%). Discontinuation rates in the two groups were similar.

**Conclusions:**

This trial revealed the clinical practice of NP and LX treatment among HER2+ MBC patients pretreated with trastuzumab in China. More patients received LX than NP in real-world while the efficacy of NP exceeded LX in terms of PFS regardless of resistant status of trastuzumab. Although the NP group had more diarrhea cases, toxicities in both groups were acceptable.

## Introduction

According to the latest epidemiological studies, breast cancer has surpassed lung cancer and has become the most common tumor type worldwide, accounting for 2.3 million new cases and 0.69 million deaths per year (1). In China, a growing number of incidence and mortality of breast cancer were observed and reached 304 thousand new diseases and 70 thousand deaths in 2015 (2).

Approximately 15–20% breast cancer patients have amplification of the human epidermal growth factor receptor-2 (HER2) expression, which is proved to be associated with aggressive biological behavior and poor clinical outcomes (3, 4). With the advent of anti-HER2 era, novel agents such as lapatinib, ado-trastuzumab emtansine (T-DM1), neratinib, and fam-trastuzumab deruxtecan-nxki (DS8201) have greatly improved the survival of HER2-positive subtype of metastatic breast cancer (HER2+ MBC); however, due to treatment resistance and drug availability, more agents are warranted in HER2+ disease.

Lapatinib is an oral, reversible, tyrosine kinase inhibitor (TKI) targeting domain of epidermal growth factor receptors (ErbBs) 1 and 2. EGF100151 trial included 399 pretreated HER2+ MBC patients and showed the result of significantly better time to progression (TTP) in lapatinib plus capecitabine (LX) group compared to capecitabine monotherapy group (p < 0.01) (5). Based on this trial, lapatinib has been approved and widely applied in later lines of treatment for HER2+ MBC.

Pyrotinib is a novel pan-ErbB irreversible receptor TKI with promising efficacy in breast cancer (6). An open-label, phase II trial indicated that pyrotinib plus capecitabine significantly extends progression-free survival (PFS) compared with LX treatment (18.1 *versus* 7.0 months, p < 0.001) (7). A phase III PHENIX study demonstrated that pyrotinib plus capecitabine has better efficacy than capecitabine monotherapy with regard to mPFS (11.1 *vs* 4.1 months, p < 0.001) (8). The phase III PHOEBE study, on the other hand, proved the results of phase II trial with the mPFS of 12.5 months in pyrotinib plus capecitabine group and 6.8 months in LX group (p < 0.0001) (9). The Chinses drug administration approved pyrotinib for use as a second line treatment for HER2+ MBC in 2020, and the clinical trial is ongoing in America.

Although capecitabine is the most common partner of pyrotinib, more chemotherapy agents need to be explored for the benefit of patients. Vinorelbine is an oral, semi-synthetic, vinca alkaloid which is approved for treatment of MBC worldwide. Vinorelbine shows efficacy and safety in combination with trastuzumab and neratinib in clinical trials (10, 11). Moreover, patients could develop capecitabine resistance before the use of pyrotinib, in which case vinorelbine is an option.

In a real-world study, vinorelbine is found to be used in nine patients treated with pyrotinib; however, detailed data are not shown (12). Furthermore, no direct comparison of pyrotinib plus vinorelbine (NP) and LX has been conducted.

Therefore, this study is carried out to uncover the real-world pattern, efficacy, and safety of NP and LX among pretreated HER2+ MBC patients, thus providing evidence for clinical practice.

## Methods

### Patients and Treatments

We screened all MBC patients treated with NP or LX between June 2015 and January 2021 from databases at five institutions, including Fudan University Shanghai Cancer Center, West China Hospital Sichuan University, Beijing San Huan Cancer Hospital, Jiangsu Province Hospital, and Shandong Cancer Hospital. The eligibility criteria were as follows: (1) pathologically confirmed HER2-positive metastatic breast cancer patients: immunohistochemical (IHC) analysis scored +3 or IHC scored +2 with a positive result of fluorescence *in situ* hybridization (FISH). (2) Patients previously received trastuzumab in the advanced setting and a taxane in any setting. (3) Patients received lapatinib (750–1,250 mg/day) plus capecitabine (1,500–2,000 mg/m^2^) or pyrotinib (320–400 mg/day) plus vinorelbine (25 mg/m^2^ intravenously or 60 mg/m^2^ orally on days 1 and 8 per 21 days) for at least one cycle, starting from June 2015 to January 2021. (4) Patients had complete medical records. All data were collected retrospectively from electronic medical record system in individual institutions. This study was approved by the Fudan University Shanghai Cancer Center Ethic Committee and Institutional Review Boards for clinical evaluation. All of the methods and analysis in this study were in accord with the Declaration of Helsinki and the relevant guidelines. This research is registered under clinicaltrials.gov (NCT 04850625).

### Outcome Measurements

The primary efficacy measure was PFS; secondary efficacy measures were OS and safety. PFS was defined as the time from treatment initiation to disease progression or death from any cause. OS was defined as the time from treatment initiation to death from any cause. Safety was evaluated as adverse events (AEs) according to the National Cancer Institute Common Terminology Criteria for Adverse Events (CTCAE) version 4.03. Tumor evaluation was defined and assessed according to the Response Evaluation Criteria in Solid Tumors (RECIST) 1.1. Disease free interval (DFI) was defined as time from surgery to diagnosis of metastasis. Definition of trastuzumab resistance was diagnosis of new recurrences during or within 12 months after adjuvant trastuzumab or confirmed progression within 3 months after first-line trastuzumab in the metastatic setting (13).

### Statistics

All patients who met the criteria were enrolled and evaluated. Descriptive statistics was used in summary of clinicopathologic characteristics, and Chi square test was used to compare the two groups. Real-world practices of therapy options were described.

PFS and OS were estimated by the Kaplan–Meier method, and the hazard ratios (HRs) and corresponding 95% confidence intervals (CIs) were estimated using the Cox proportional hazard model. Subgroup analysis was performed using the Cox regression model and shown by forest plot. Cox multivariate models were performed based on the univariate analyses results. P-value less than 0.05 was considered statistically significant. Statistical analyses were performed using SPSS version 23. Forest map was completed by Graphpad Prism version 7.0.

## Results

### Patients and Treatments

The medical records of all consecutive patients using LX/NP who met our criteria were retrospectively reviewed, and 224 patients were enrolled and evaluated in this trial.

Among the 224 patients, 132 (58.9%) patients received lapatinib plus capecitabine and 92 (41.1%) patients received pyrotinib plus vinorelbine. Baseline patient characteristics between two treatment groups were summarized in **Table 1**. The median age of both groups was 52 years, ranging from 26 to 86 for LX group and 26 to 74 for NP group. A majority of patients received surgery while 16 and 15% patients were *de novo* stage IV in LX and NP groups, respectively. Visceral metastasis accounted for 26% patients in LX and 30% in NP. The median prior treatment lines for metastatic disease are two in both groups. In both groups, more patients had trastuzumab refractoriness than trastuzumab resistance. Overall, no significant differences were observed in baseline status between the two groups.

**Table 1:** Baseline characteristics of patients grouped by LX or NP.

Characteristics	LX N = 132 n (%)	NP N = 92 n (%)	P-values
Median age(range)	52 (26–86)	52 (26–74)	0.432
DFI			
<2 years	58 (44)	29 (32)	0.122
≥2 years	53 (40)	49 (53)	
*de novo* stage IV breast cancer	21 (16)	14 (15)	
Number of metastatic sites			
1	53(40)	26 (28)	0.171
2	35(26)	27 (29)	
≥3	44(33)	39 (42)	
Visceral disease			
Yes	35 (26)	28 (30)	0.548
No	97 (73)	64 (70)	
ER/PR status			
Positive	69 (52)	40 (43)	0.462
Negative	63 (48)	52 (57)	
Median no. of prior treatment of metastatic disease (range)	2 (1–6)	2 (1–4)	0.734
Trastuzumab resistance status			
Resistance	29 (22)	30 (33)	0.119
Refractoriness	93 (70)	57 (62)	
Not known	10 (8)	5 (5)	

### Treatment Efficacy

**Figure 1:**
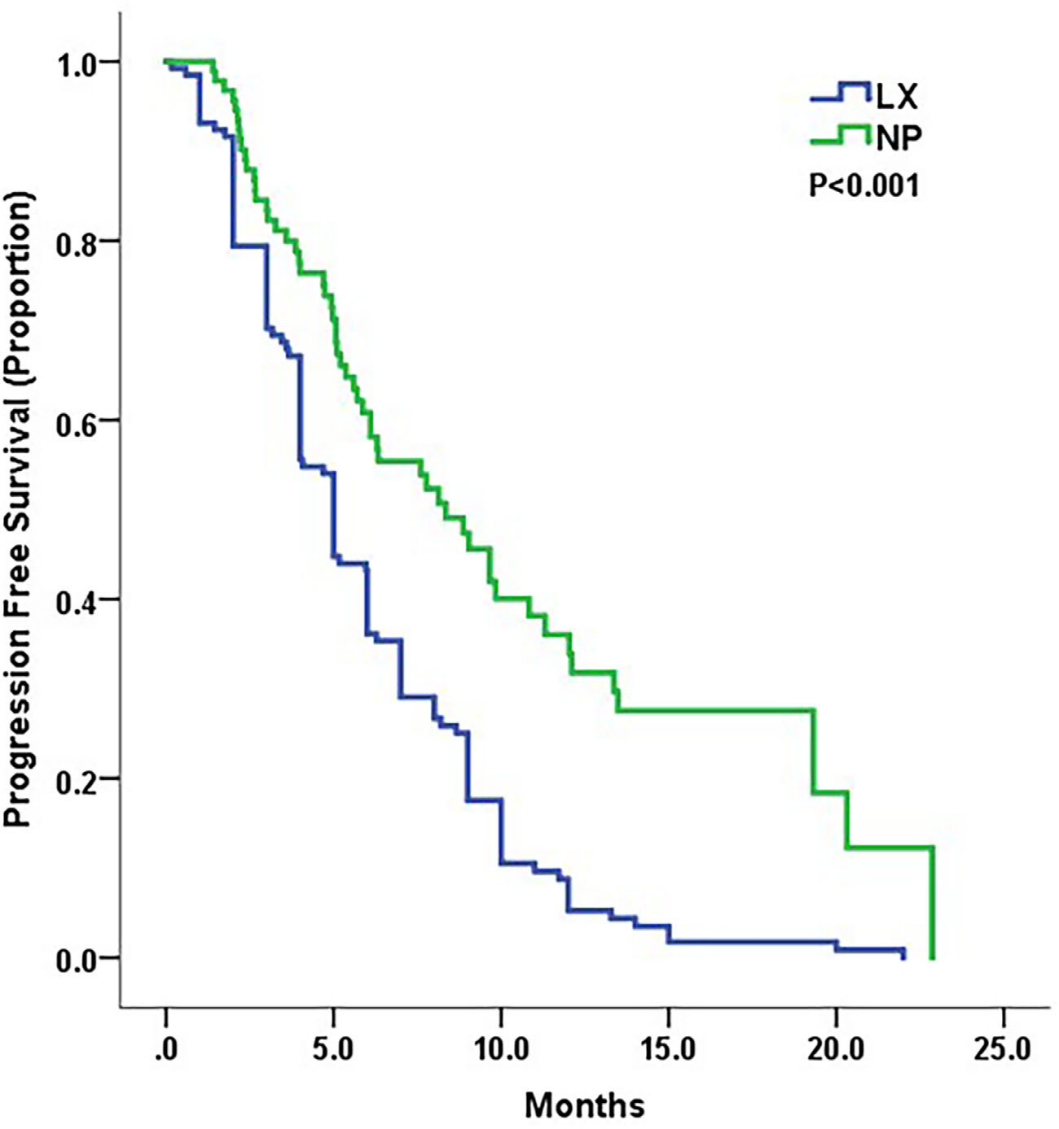
Kaplan–Meier curves for progression-free survival by treatment arm.

With a median 20-month follow-up, 126 of 132 patients in LX and 57 of 92 patients in NP experienced progressive disease (PD). The median PFS of NP group was 8.3 months and of LX group was 5.0 months. NP group showed a significant improvement in PFS compared to LX (HR 0.47, 95% CI, 0.34–0.65, p < 0.001, **Figure 1**). The median OS was not reached at the time of analysis.

**Figure 2:**
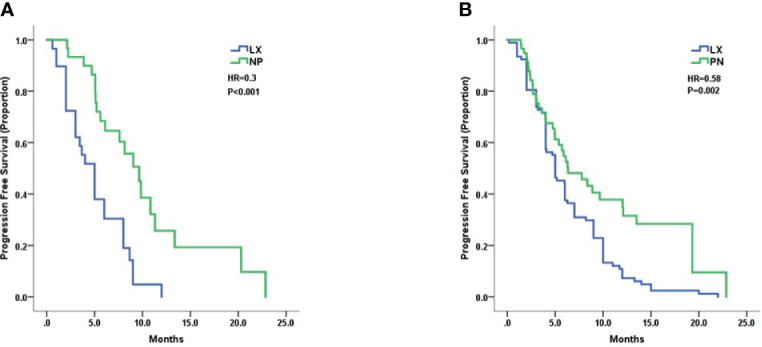
Kaplan–Meier curves for progression-free survival by treatment arm for patients with: **(A)** trastuzumab resistance and **(B)** trastuzumab refractoriness.

Treatment efficacy of LX and NP grouped by trastuzumab resistant status was analyzed. Patients with trastuzumab resistance had a mPFS of 5.0 months in LX group and 9.6 months in NP group (HR 0.30, 95% CI 0.16–0.57, p < 0.001, **Figure 2A**). Patients with trastuzumab refractoriness had a mPFS of 5.0 months in LX group and 6.3 months in NP group (HR 0.58, 95% CI 0.39–0.86, p = 0.004, **Figure 2B**).

**Figure 3:**
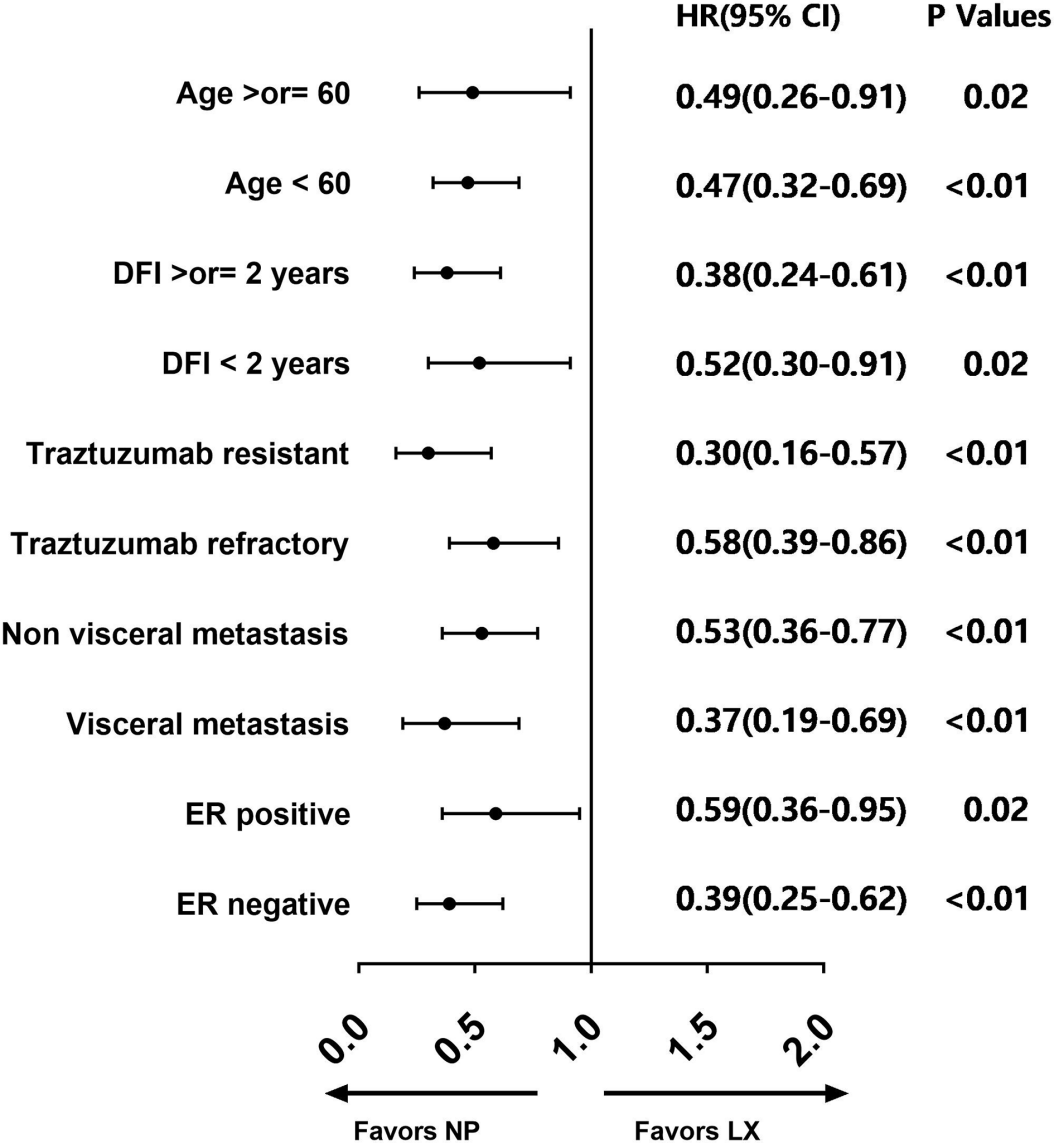
Forest plot for subgroup analysis.

In subgroup analysis, the PFS advantage of NP over LX was observed in most subgroups, including different age groups, DFI length, visceral metastasis status, and ER/PR status. The forest plot of subset analysis was shown in **Figure 3**.

In terms of univariate analysis, NP therapy (HR 0.47, 95% CI 0.34–0.65 p < 0.001) was a predictive factor of longer PFS, while more metastatic sites (HR 1.09, 95% CI 1.00–1.41 p = 0.048) and more prior MBC treatment lines (HR 1.16, 95% CI 1.03–1.31 p = 0.016) were risk factors for PFS. With regard to multivariate analysis, NP treatment (adjusted HR 0.44, 95% CI 0.32–0.63, p < 0.001) emerged as independent prognostic factors even after balancing the ER/PR status, DFI, age, visceral metastasis, number of metastatic sites, prior MBC treatment lines, and trastuzumab resistant status. More metastatic sites (adjusted HR 1.25, 95% CI 1.04–1.51, p = 0.017) and more prior MBC treatment lines (adjusted HR 1.18, 95% CI 1.05–1.32, p = 0.004) were independent risk factors of progression.

### Safety

Grade 3 or 4 adverse events were collected in **Table 2**. NP group had more diarrhea cases (23.9%) compared to LX group (8.3%), while LX group occurred more palmar-plantar erythrodysesthesia syndrome (4.5 *vs* 0%) and increased alanine aminotransferase (2.3 *vs* 0%). Two groups had similar hematologic toxicities and fatigue. Nine patients (9.8%) in NP and ten patients (7.8%) in LX withdrew treatment because of toxicities, and other patients continued treatment. All adverse events were reversed after symptomatic treatment, and no patient died of toxicity. Overall, both treatment regimens presented an acceptable safety.

**Table 2:** Adverse events (grade 3/4).

Adverse Events (grade 3/4)	NP N = 92 n (%)	LX N = 132 n (%)
Diarrhea	22 (23.9)	11 (8.3)
Neutropenia	7 (7.6)	7 (5.3)
Leukopenia	4 (4.3)	10 (7.6)
Anemia	2 (2.2)	1 (0.8)
Thrombocytopenia	1 (1.1)	1 (0.8)
Nausea and vomiting	1 (1.1)	1 (0.8)
Fatigue	1 (1.1)	2 (1.5)
Weight loss	1 (1.1)	0
Palmar-plantar erythrodysesthesia syndrome	0	6 (4.5)
Alanine aminotransferase increased	0	3 (2.3)
Dizziness	0	1 (0.8)
All	39	43

## Discussion

This study revealed the real-world clinical practice of lapatinib plus capecitabine *versus* pyrotinib plus vinorelbine in HER2+ MBC patients after failure of trastuzumab and taxane. Previously, we evaluated the efficacy and safety of NP alone (14). To the best of our knowledge, this is the first investigation in the comparison between LX and NP.

Firstly, more patients received traditional LX treatment (58.9%) than NP regimen (48.1%). This phenomenon indicated that although both regimens are commonly used in daily practice, LX treatment was more favored in HER2-positive MBC patients pretreated with trastuzumab. The possible reason could be: pyrotinib was approved and produced later than lapatinib; NP had a higher price than LX; lack of evidence of NP treatment. A real-world study performed in Europe showed that among 451 HER+ MBC patients, 236 (53%) patients received trastuzumab, 220 (52%) were treated with pertuzumab and trastuzumab, 79 (19%) with lapatinib, and 108 (28%) with T-DM1 (15), which were mainly attributed to the standard use of trastuzumab in first line setting. It is noteworthy that T-DM1 was not available in China until recent.

Above all, our study revealed the superior efficacy of NP over LX in HER2-positive metastatic breast cancer patients pretreated with trastuzumab and taxane considering the significantly improved PFS.

Previous study has proved the efficacy and safety of LX. EGF100151 study enrolled 399 HER2+ MBC patients pretreated with trastuzumab and indicated a significantly improved mTPP of 6.0 months of LX group compared to X arm (p < 0.01) (5). LX was widely recommended and used in later lines for HER2+ MBC patients. PFS data in our study was consistent to that of randomized controlled trials (RCTs).

Pyrotinib is a promising novel oral TKI approved in China. In a phase I trial, HER2+ MBC patients received pyrotinib with escalated dose and the overall response rate was 50.0% (18 of 36), with a 35.4-week mPFS (95% CI, 23.3 to 40.0 weeks) (16). A multicenter, randomized phase II study enrolled 128 patients pretreated with taxanes, anthracyclines, and/or trastuzumab and randomized into capecitabine combined with lapatinib or pyrotinib, and results showed that compared to lapatinib plus capecitabine, pyrotinib plus capecitabine significantly improved the ORR (78.5 *vs* 57.1%, p = 0.01) and PFS (mPFS 18.1 *vs*. 7.0 months, p < 0.001) (7). A double-blinded, multicenter, randomized phase III PHENIX study included patients pretreated with trastuzumab and taxanes and reported that pyrotinib plus capecitabine had better PFS than capecitabine monotherapy (mPFS 11.1 *vs* 4.1 months, p < 0.001) (8). We reported a mPFS of 8.3 months in the NP group, which was slightly lower than those of RCT; the reason might be in our study, patients received more previous treatment lines and were less sensitive to anti-HER2 treatment.

With regard to other novel anti-HER2 treatments, T-DM1, DS-8201, and neratinib were potential candidates for pretreated HER2+ MBC. In a phase III EMILIA study, 991 pretreated HER2-positive metastatic breast cancer patients were randomized to T-DM1 group and LX group, and the results demonstrated that T-DM1 group had significantly better PFS than LX group (mPFS 9.6 *vs* 6.4 months, P < 0.01) (17). Although T-DM1 had similar mPFS compared with NP in our study, the incidence of severe thrombocytopenia and high price limited its use. A phase III NALA trial enrolled 621 MBC patients previously treated with two or more anti-HER2 therapy and found that neratinib plus capecitabine had prolonged PFS than LX group (mPFS 8.8 *vs* 6.6 months, p < 0.001) (18). We observed a similar efficacy regarding NP in our study. Another antibody–drug conjugate (ADC), DS-8201, was focused because of its remarkable efficacy. In a phase II DESTINY-Breast01 trial, 184 HER2+ MBC patients with a median of six prior treatments received DS-8201 and results showed a mPFS of 16.4 months, with an ORR of 60.9% (19). Despite the excellent data from phase II trial, further investigation of overall survival and more convenient availability is warranted for DS-8201. The summary and comparison of RCTs were shown in **Table 3**. In all, NP regimen in our study was no less than T-DM1 and neratinib with regard to PFS.

**Table 3:** Trials of novel regimens in second line treatment for HER2-positive MBC.

Trial/Year	Phase	Previous	Treatment	Size	TTP/PFS (m)	OS (m)
EMILIA/2013	III	trastuzumab and taxane	LX	496	6.4 p < 0.001	25.1 p < 0.001
			T-DM1	495	9.6	30.9
PHENIX/2020	III	trastuzumab and taxane	X	94	4.1 p < 0.001	NA
			Pyrotinib+X	185	11.1	
NALA/2020	III	≥2 prior anti-HER2 treatment	LX	314	6.6 p = 0.006	NA
			Niratinib + X	307	8.8	
DESTINY-Breast01/2020	II	T-DM1	DS8201	184	16.4	NA
This study/2021	Real-world	trastuzumab and taxane	LX	132	5.0 p < 0.001	NA
			NP	92	8.3	

Our trial indicated that the prolonged PFS of NP group was regardless of trastuzumab resistant status, which conformed to phase III study (8). This phenomenon, on the other hand, partly demonstrated the reversing effect of pan-HER2 TKI on trastuzumab-resistance, which could be attributed to the blockage of HER1 and HER4 receptors except HER2, thus bypassing the possible resistant pathway.

Moreover, in an exploratory subgroup analysis, the advantage of NP regimen was maintained in all preset subgroups, including age < or ≥60, DFI < or ≥2 years, visceral metastasis or not, ER/PR positive or negative, which further demonstrated the superior efficacy of NP over LX.

Multivariate analysis revealed more metastatic sites and more prior treatment lines as independent risk factors for PFS. More metastatic sites usually suggested a heavier tumor burden, leading to poor drug response and survival. Prior treatment lines indicated use of different anti-HER2 treatment as well as chemotherapy, which could result in cross-talk drug resistance.

In terms of toxicity, both agents were tolerated. The most common grade 3/4 AE in the NP group was diarrhea (23.9%) compared to the LX group (8.3%), which was accorded with a report of 15.4–30.8% in previous study of pyrotinib (7, 8). Diarrhea could be reversed with routine loperamide or montmorillonite treatment. If diarrhea continued, both vinorelbine and pyrotinib were interrupted until diarrhea resolved to grade 0 to 1. Dose reduction was applied if necessary. LX group experienced palmar-plantar erythrodysesthesia syndrome and abnormal liver function, which were possibly related to capecitabine. Oily moisturizer and topical cortisol were applied for palmar-plantar erythrodysesthesia syndrome, and hepatoprotective drugs were used for impaired hepatic function.

In conclusion, this trial revealed the clinical practice of NP and LX treatment in HER2+ MBC patients previously treated with trastuzumab and taxane in China. More patients received LX than NP in real-world, while the efficacy of NP exceeded LX in terms of PFS regardless of resistant status of trastuzumab. Although the NP group had more diarrhea cases, toxicities in both groups were acceptable.

Since this study is retrospective, RCT is needed for further evidence. Several ongoing trials are exploring pyrotinib in first-line MBC treatment, neo-adjuvant and adjuvant treatments for early stage breast cancer. With the development of targeted therapy in HER2-positive breast cancer, physicians have more choices while the sequential use and choice of anti-HER2 therapy should be carefully evaluated for the best benefit of patients.

## Conclusions

This trial revealed the clinical practice of NP and LX treatment in HER2+ MBC patients previously treated with trastuzumab and taxane in China. More patients received LX than NP in real-world, while the efficacy of NP exceeded LX in terms of PFS regardless of the resistant status of trastuzumab. Although the NP group had more diarrhea cases, toxicities in both groups were acceptable.

## Data Availability Statement

The raw data supporting the conclusions of this article will be made available by the authors, without undue reservation.

## Ethics Statement

All of the methods and analysis in this study involving human participants accorded with the ethical standards of research committee and the 1964 Helsinki declaration and its later amendments or comparable ethical standards. The study has been approved from Fudan University Shanghai Cancer Center Ethic Committee and Institutional Review Boards.

## Author Contributions

YX collected all of the data from database, performed statistical analysis, and finished the manuscript. YL participated in the manuscript. LT, DS, PY, WL, and HL provided data from different centers. RG and BW designed, carried out the study and revised the manuscript. All authors contributed to the article and approved the submitted version.

## Conflict of Interest

The authors declare that the research was conducted in the absence of any commercial or financial relationships that could be construed as a potential conflict of interest.

The handling editor declared a shared affiliation, though no other collaboration, with one of the authors PY at the time of the review.

## Publisher’s Note

All claims expressed in this article are solely those of the authors and do not necessarily represent those of their affiliated organizations, or those of the publisher, the editors and the reviewers. Any product that may be evaluated in this article, or claim that may be made by its manufacturer, is not guaranteed or endorsed by the publisher.

## Abbreviations

MBC, metastatic breast cancer; HER2, human epidermal growth factor receptor 2; OS, overall survival; HR, hazard ratio; CI, confidence interval; NCCN, National Comprehensive Cancer Network; RECIST, Response Evaluation Criteria in Solid Tumors; DFI, disease-free interval; CTCAE, Common Terminology Criteria for Adverse Events; SPSS, Statistical Product and Service Solutions.
